# Tics in patients with encephalitis

**DOI:** 10.1007/s10072-021-05065-w

**Published:** 2021-01-23

**Authors:** James Badenoch, Tamara Searle, Iona Watson, Andrea E. Cavanna

**Affiliations:** 1 Department of Neuropsychiatry, BSMHFT and University of Birmingham, Birmingham, UK; 2 School of Life and Health Sciences, Aston University, Birmingham, UK; 3 University College London and Institute of Neurology, London, UK

**Keywords:** Encephalitis, Gilles de la Tourette syndrome, Hyperkinetic movement disorders, Tics

## Abstract

**Background:**

Movement disorders have been described in the context of different types of encephalitis. Among hyperkinetic manifestations, tics have sporadically been reported in cases of encephalitis resulting from a range of aetiologies.

**Objective:**

This review aimed to assess the prevalence and characteristics of tics in patients with encephalitis.

**Methods:**

We conducted a systematic literature review of original studies on the major scientific databases, according to the standards outlined in the Preferred Reporting Items for Systematic Reviews and Meta-Analyses (PRISMA) guidelines.

**Results:**

In addition to the established association between tics and encephalitis lethargica, our literature search identified reports of tics in patients with immune-mediated pathologies (including autoimmune encephalitides affecting the *N*-methyl-d-aspartate receptor, voltage-gated potassium channels, and glycine receptors) and infective processes (ranging from relatively common viral pathogens, such as herpes simplex, to prions, as in Creutzfeldt-Jakob disease). Tics were most commonly reported in the post-encephalitic period and involvement of the basal ganglia was frequently observed.

**Discussion:**

The association of new-onset tics and encephalitis, in the background of other neuropsychiatric abnormalities, has practical implications, potentially improving the detection of encephalitis based on clinical features. Future research should focus on the categorisation and treatment of hyperkinetic movement disorders associated with encephalitis.

## Introduction

Encephalitis can be the result of a post-infectious process as well as an autoimmune reaction [1]. Multiple neuropsychiatric presentations of encephalitis have been described, encompassing a range of movement disorders [2]. Movement disorders as a result of encephalitis include chorea, myoclonus, dystonic jerks, and other hyperkinetic manifestations [2]. Moreover, encephalitis has long been associated with the onset of tics. Tics are currently defined as a sudden, rapid, recurrent, nonrhythmic movements or vocalisations [3]. Tics typically develop in early life as the key feature of a primary tic disorder such as Giles de la Tourette syndrome (GTS), a neuropsychiatric condition characterised by the presence of multiple motor and phonic tics, frequently associated with behavioural problems. The onset of tics in adults is unusual and is often related to an underlying brain pathology, such as the neurodegenerative process that characterises Huntington disease [4]. Case series have also documented the development of spontaneous tics in patients following trauma and drug exposure [5]. Although hyperkinetic movement disorders have been commonly associated with encephalitis, there has been no review to date analysing the presence of secondary tics in this heterogeneous patient population.

## Methods

The present systematic literature review was conducted in accordance with the Preferred Reporting Items for Systematic Reviews and Meta-Analyses (PRISMA) guidelines [6], used in conjunction with the Explanation and Elaboration document [7]. The searches were conducted on five databases: MEDLINE, Embase, Ovid, PsycInfo, and PsycArticles. The search terms were as follows: ‘Tic*’ AND ‘Encephalit*’. For comprehensiveness, the reference lists of eligible articles were also screened to identify any relevant articles. We limited our search to original studies and case reports/series published in the English language, but there were no chronological, geographic, or demographic limitations to the inclusion of articles.

## Results

**Fig. 1:**
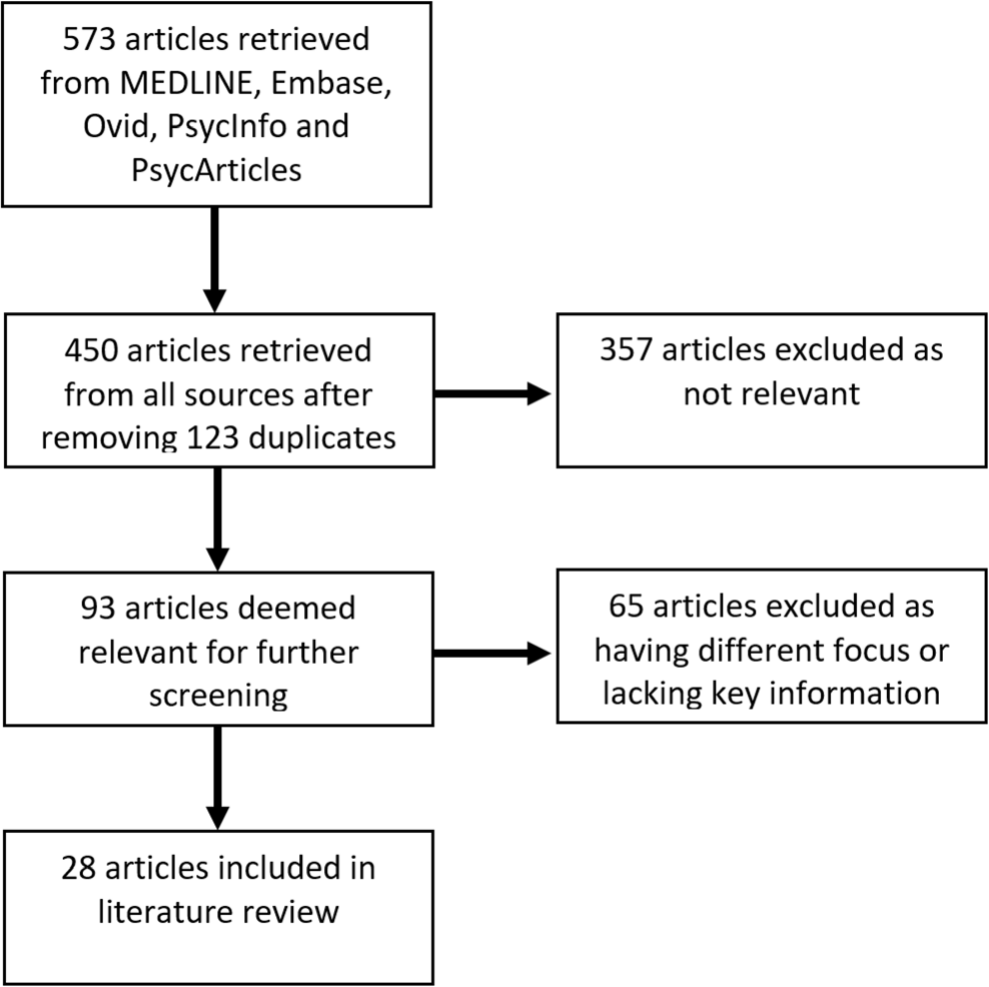
PRISMA flow diagram outlining the identification, screening, assessment for eligibility, and inclusion of studies in the present review

Our systematic literature search yielded a total of 450 articles, after removal of duplicates. Of these, 93 were considered relevant to the review and their full texts were inspected. A further 65 studies were excluded because they had a different focus or lacked key information. A total of 28 articles were included in the present review. The article selection process is summarised in the PRISMA flow diagram (Fig. 1).

The reviewed articles include both retrospective cohort studies/case series and individual case reports of patients who presented with tics in the context of three groups of encephalopathies: encephalitis lethargica (Tables 1 and 2), autoimmune encephalitides (Tables 3 and 4), and post-infectious encephalitides (Tables 5 and 6).

**Table 1:** Summary of retrospective cohort studies and case series of patients with encephalitis lethargica and tics

Study	Country	Number	Mean age (range)	Male (%)	Aetiology of encephalitis	Tics	Other neuropsychiatric symptoms	Additional findings	Main limitations
Calne et al. [8]	UK	40	47–73	45	Encephalitis lethargica	3 patients developed tics	Parkinsonism reported as very common; 9 patients with psychiatric disturbances (6 aggressive and uncooperative, 1 obsessional, 1 paranoid, 1 depressed); 4 with choreoathetosis	All patients had a history of illness in the 1917 encephalitis pandemic and had been in a hospital for an average of 29 years	Case histories not documented for individual patients; description of tics lacking detail
Sacks et al. [9]	USA	25	N/A	N/A	Encephalitis lethargica	8 patients developed respiratory and phonic tics (sudden deep breaths, yawns, coughs, giggles, sighing, grunting, moaning)	Severe post-encephalitic syndrome, with the development of respiratory crises and tics after treatment with l-dopa	In some cases (not quantified), the respiratory tics and breathing abnormalities were present before the administration of l-dopa and increased after its use	Lack of detail about the presence of tics before the administration of l-dopa and its effects on individual patients
Dale et al. [10]	UK	20	2–69	55	Basal ganglia encephalitis, reported as encephalitis lethargica	2 patients developed tics: a 10-year-old boy had motor tics; a 17-year-old boy had both motor and phonic tics	Hypersomnolence, bradykinesia, mutism, and depression (10-year-old boy); sleep inversion, bradykinesia, stereotypies, mutism, catatonia, obsessive-compulsive disorder, trichotillomania (17-year-old boy)	N/A	Description of tics lacking detail (including data on tic onset)
Dale et al. [11]	Australia	17	7 (0–15)	53	Basal ganglia encephalitis, reported as encephalitis lethargica	1 patient developed motor tics	Somnolence, lethargy, parkinsonism	Diagnosis based on the presence of somnolence, an akinetic movement disorder, and emotional dysregulation in the background of basal ganglia changes on neuroimaging	Demographic data of individual patients not reported; description of tics lacking detail

**Table 2:** Summary of individual case reports of patients with encephalitis lethargica and tics

Study	Country	Age	Sex	Aetiology of encephalitis	Tics	Other neuropsychiatric symptoms	Additional findings	Main limitations
Russel [12]	Canada	23	F	Encephalitis lethargica	Described as ‘rhythmical tic-like contraction of the left arm drawing it up across the chest and accompanied by a sort of a kicking movement of the left leg’	Sudden onset twitching of the hands and face, ocular nystagmus, involuntary micturition (in the context of chronic parkinsonism)	N/A	Lack of immunological testing
Martino et al. [13]	N/A	‘Middle-aged’	F	Encephalitis lethargica-like variant of autoimmune basal ganglia encephalitis	Unspecified tics developed after acute encephalitic episode	Acute presentation: diplopia, headache, behavioural symptoms, hypersomnolence, followed by oculogyric crises, parkinsonism, dystonic posturing of the right arm; chronic sequelae: obsessive-compulsive behaviours, anxiety, panic attacks, dysthymia	Anti-basal ganglia antibodies and striatal changes during the acute encephalitic crisis	Description of tics lacking detail
Maranis et al. [14]	Greece	28	F	Encephalitis lethargica	Unspecified orofacial tics	Acute presentation: generalised tonic-clonic seizure with subacute expressive aphasia, anxiety, and agitation; chronic sequelae: parkinsonism, delusions, emotional lability, insomnia, and catatonia	Rapid and sustained improvement with immunotherapy	Diagnosis based on clinical features (sleep disorder, parkinsonism, and psychiatric disturbance); lack of thorough autoimmune panel
Pawela et al. [15]	Australia	5	M	Autoimmune basal ganglia encephalitis described as encephalitis lethargica	Pre-existing motor tics that worsened during the encephalitis period	Acute presentation: disinhibited behaviour, mutism, dystonia, and akinesia; chronic sequelae: anxiety and obsessive-compulsive behaviours	Positive serum dopamine-2-receptor autoantibodies	Description of premorbid neuropsychiatric state lacking detail

**Table 3:** Summary of retrospective cohort study of patients with autoimmune encephalitis and tics

Study	Country	Number	Mean age (range)	Male (%)	Aetiology of encephalitis	Tics	Other neuropsychiatric symptoms	Additional findings	Main limitations
Tajudin et al. [16]	Malaysia	11	7	45	Anti-NMDAR encephalitis	2 patients developed dystonic tics	Temper tantrums, affect lability, refractory status epilepticus, hemichorea, sleep inversion, and speech impairment	Herpes simplex antibodies in 1 patient; non-specific neuroimaging abnormalities in most patients (suggestive of an infective process in 2 patients)	Description of immunological tests and motor presentations of individual patients lacking detail

*NMDAR N*-methyl-d-aspartate receptor

**Table 4:** Summary of individual case reports of patients with autoimmune encephalitis and tics

Study	Country	Age	Sex	Aetiology of encephalitis	Tics	Other neuropsychiatric symptoms	Additional findings	Main limitations
Berkeley and Sohoni [17]	USA	11	F	Anti-NMDAR encephalitis associated with an ovarian teratoma	Twitching of the face and production of ‘sharp’ and ‘barky’ sounds	Altered mental status and movements, confusion, dysarthria	Left frontal headache 2 days prior to presenting in the Emergency Department	Description of movement disorders lacking detail
Dhamija et al. [18]	USA	5	M	VGKC encephalitis	Motor tics	N/A	Neuroimaging abnormalities (left thalamus)	Limited generalisability of findings
Tajul et al. [19]	Malaysia	13	M	VGKC encephalitis	Cervical tics	Bradykinesia, choreoathetosis (upper limbs), ballismus (lower limbs), facial dystonia	Mild flu-like illness 2 weeks prior to developing behavioural changes, language difficulties, amnesia	Limited generalisability of findings
Safadieh and Dabbagh [20]	Lebanon	4	F	Anti-NMDAR encephalitis	Phonic tics	Orofacial dyskinesias, gait problems	Autoimmune neurological disorder	Limited generalisability of findings
Stern et al. [21]	UK	40	M	Anti-GlyR encephalitis	Motor tics	Affective symptoms, hallucinations	Presumed upper respiratory tract infection 5 days prior to developing behavioural changes	Description of tics lacking detail; uncertain time course (acute encephalitis or post-encephalitic reaction)
Alvarez et al. [22]	Peru	72	F	VGKC encephalitis	Orofacial tics	Hypertonia, dysmetria, echolalia, visual and auditory hallucinations, depression, asthenia, memory problems	Neuroimaging abnormalities (occipital and medial frontal cortex, mainly in the right hemisphere)	Limited generalisability of findings
Swayne et al. [23]	Australia	45	F	Anti-GlyR encephalitis	Tics	Hemiballismus (right side)	Neuroimaging abnormalities (right cerebellar hemisphere)	Description of tics lacking detail

*NMDAR N*-methyl-d-aspartate receptor, *VGKC* voltage-gated potassium channels, *GlyR* glycine receptor

**Table 5:** Summary of retrospective cohort studies and case series of patients with post-infectious encephalitis and tics

Study	Country	Number	Mean age (range)	Male (%)	Aetiology of encephalitis	Tics	Other neuropsychiatric symptoms	Additional findings	Main limitations
Richter and Shimojyo [24]	USA	68	N/A	N/A	Japanese encephalitis	7 patients developed tics	Choreoathetosis associated with tics	Late sequelae of Japanese encephalitis (5 years after initial infection)	Description of tics lacking detail
Berthier et al. [25]	Spain	13	36	63	Post-encephalitis hydrocephalus	5 patients developed motor and phonic tics	Obsessive-compulsive disorder	Differences between acquired obsessive-compulsive disorder (13 patients), idiopathic obsessive-compulsive disorder (25 patients), and controls (13 healthy participants)	Focus on obsessive-compulsive disorder rather than tics; link between tics and post-encephalitis hydrocephalus lacking detail
Mirsattari et al. [26]	Canada	6	32–38	N/A	Human immunodeficiency virus encephalitis	1 patient developed motor/facial tics	Choreoathetosis, dystonic limb posturing, spasmodic torticollis	First presentation of human immunodeficiency virus	Diagnosis of encephalitis lacking detail
Francisco et al. [27]	USA	33	12	81	West Nile virus encephalitis	1 patient (5-year-old boy) developed facial tics 3 weeks after recovery from acute viral illness	Athetosis at the same time as tic onset; decreased movements of the limbs, cognitive decline, bladder and bowel incontinence, speech and swallowing problems	Immunosuppressed patients	Focus on West Nile virus rather than tics or encephalitis
Mejia and Jankovic [28]	USA	155	41	65	Rubella encephalitis, *Mycoplasma pneumoniae* encephalitis	1 patient (8-year-old girl with rubella encephalitis) + 1 patient (18-year-old man with *Mycoplasma pneumoniae* encephalitis) developed motor and phonic tics	Gait problems, ataxia	Neuroimaging abnormalities (basal ganglia)	Link between neuroimaging abnormalities and tics lacking detail; diagnosis of encephalitis lacking detail
Teke et al. [29]	Turkey	7	12 (5–15)	71	Neurobrucellosis	1 patient (11-year-old boy) developed phonic tics (coughing)	N/A	Initially diagnosed as a pulmonary manifestation of brucellosis	Description of tics lacking detail

**Table 6:** Summary of individual case reports of patients with post-infectious encephalitis and tics

Study	Country	Age	Sex	Aetiology of encephalitis	Tics	Other neuropsychiatric symptoms	Additional findings	Main limitations
Northam and Singer [30]	USA	6	F	Herpes simplex encephalitis	Eye blinking, grimacing, head twitching; complex motor and phonic tics (2 weeks after recovery)	Malaise, headache, seizures, nausea, and vomiting	Tics improved with antidopaminergic medication	Diagnosis of encephalitis lacking detail
Dale et al. [31]	Australia	4	M	Varicella zoster encephalitis	Chronic motor tics affecting face and neck (2 months after onset)	Alterations of consciousness and tremor	Neuroimaging abnormalities (basal ganglia)	Link between neuroimaging abnormalities and tics lacking detail
Sabuncuoğlu and Berkem [32]	Turkey	9	M	Mumps—meningoencephalitis	Eye blinking, finger flexing, throat clearing (2 months after recovery)	N/A	Tics improved with antidopaminergic medication	Link between mumps and tics lacking detail
Molina and Feteke [33]	USA	59	F	Creutzfeldt-Jakob disease	Grunting and barking (5 months after onset)	Involuntary movements, akinetic mutism, catatonia, personality change	Patient was mute except for phonic tics	Limited generalisability of findings
Dey and Bhattacharya [34]	India	10	M	Measles—subacute sclerosing panencephalitis	Insidious onset complex shoulder and facial tics	Ataxia and myoclonus	Tics as presenting symptoms	Limited generalisability of findings
Anandatia et al. [35]	Philippines	28	F	Toxoplasma encephalitis secondary to human immunodeficiency virus	Motor/facial tics	Hemichorea and alterations of consciousness	N/A	Limited generalisability of findings

## Discussion

### Encephalitis lethargica

Encephalitis lethargica was first described in 1917 by Austrian neurologist Constantin von Economo in a clinically and pathologically heterogeneous cohort of patients presenting with a lethargic state in the context of significant neuropsychiatric disturbances [36]. Although the term ‘encephalitis lethargica’ was first used by von Economo with reference to the pandemic that spread globally between 1917 and 1929, similar clinical pictures had previously been described during other widespread pathologies, such as the ‘nona’ epidemic that appeared in northern Italy in 1890 [4].

The aetiology of the encephalitis lethargica pandemic is still disputed. The main theories that have been proposed include an invasive influenza encephalitis triggered by the twentieth century Spanish influenza, an environmental toxin, and an autoimmune basal ganglia disease [37, 38]. Tics arising on the background of an immune-mediated basal ganglia pathology suggest an interesting parallel with research demonstrating basal ganglia dysfunction in GTS and a role for autoimmunity in the pathophysiology of tics [39]. In addition to the historical descriptions from the 1917 pandemic, there have been sporadic reports of tics in patients with encephalitis lethargica throughout the following century.

#### The 1917 pandemic

The clinical phenotype of patients with encephalitis lethargica during the pandemic was highly variable between the acute encephalitic state and the post-encephalitic syndrome, which may be separated by a period of symptom resolution. Several attempts have been made to categorise the symptoms into distinct clinical subtypes [8;12;37]. Von Economo described three clinical subtypes: a hypersomnolent-ophthalmoplegic form, an amyostatic-akinetic form, and a hyperkinetic form [4;37].

Although the cases documented during the epidemic presented with involuntary movements in both the acute and post-encephalitic phases, the wide spectrum of hyperkinetic manifestations, encompassing tics and chorea, has received relatively little attention. In addition to chronic parkinsonism, the post-encephalitic sequelae included shouting episodes resembling phonic tics that were termed ‘klazomania’ and were frequently associated with oculogyric spasms in adults. In children, both tics and behavioural problems consistent with attention-deficit and hyperactivity disorder (a common co-morbidity of GTS) tended to persist despite the resolution of other symptoms [37].

Sacks’ detailed description of post-encephalitic symptoms in 20 patients encompassed a range of neuropsychiatric symptoms developed decades after the acute phase [40]. Sacks reported a wide range of simple and complex tics, such as coprolalia and arithmomania [41], characteristically preceded by premonitory urges that had been inconsistently described in other sources. In addition to highlighting an association between tics, oculogyric crises, and catatonia [42], Sacks found that a number of patients experienced ‘tic attacks’, often in response to environmental triggers and emotional states—as documented in GTS [43]. Based on Sacks’ description, it is possible that the administration of levo-dopa led to an increase in the severity of pre-existing tics in a number of patients. The exacerbation of tics in patients receiving levo-dopa is in keeping with the pathophysiological model of tics as associated with excess of dopaminergic neurotransmission, as well as with the widespread observations of tic improvement in patients receiving antidopaminergic agents [39, 43].

#### More recent cases

There have been sporadic reports of encephalitis lethargica cases since the pandemic. However, a lack of consensus regarding its aetiology has resulted in the condition being given different labels. Dale et al. reported two case series of children with basal ganglia encephalitis, which they refer to as recent cases of encephalitis lethargica [11, 38]. The criteria used to diagnose basal ganglia encephalitis included somnolence, movement disorder, and emotional dysregulation in the background of neuroimaging changes affecting the basal ganglia [11, 38]. The first series of 20 patients was characterised by high clinical heterogeneity, with somnolence, lethargy, and parkinsonism as the most common findings [11]. Chronic neuropsychiatric impairment was present in half of the patients, with rarer cases of oculogyric crisis [44]. Two patients were reported to have tics (without details on the time course of their development): a 10-year-old boy with hypersomnolence, bradykinesia, and motor tics, and a 17-year-old boy with sleep inversion, bradykinesia, and multiple motor and phonic tics [10]. The diagnostic criteria used by Dale to define basal ganglia autoimmune encephalitis was different to those used by von Economo to define encephalitis lethargica. Interestingly, the results of a follow-up study revealed that half of the patients had NMDAR-positive autoantibodies, raising the possibility of heterogeneous pathophysiological pathways [45]. Overall, the lack of available immunological testing and neuroimaging resources at the time of the pandemic creates difficulty when attempting to compare more recent cases with the initial reports [4]. In Dale et al.’s later case series of 17 patients with presumed encephalitis lethargica, only one had tics: the demographic and clinical characteristics of the individual patients were not reported [11].

A case report by Martino et al. described an encephalitis lethargica-like variant of autoimmune basal ganglia encephalitis with serum-positive anti-basal ganglia antibodies [13]. The patient was a middle-aged woman with an acute encephalitis presenting with hypersomnolence, followed by tics in the context of a profoundly parkinsonian post-encephalitic state [13]. A case report by Maranis et al. described a 28-year-old woman who presented with a generalised tonic-clonic seizure on the background of subacute dysphasia [14]. The motor manifestations included orofacial tics and parkinsonism. The diagnosis of encephalitis lethargica was based on the presence of sleep disorder and psychiatric disturbances, in addition to parkinsonism.

Other reports of autoimmune basal ganglia encephalitis, described as more recent cases of encephalitis lethargica, have focused on the presence of tics and other dyskinesias within the hyperkinetic phenotype defined by von Economo. A case series of four patients with basal ganglia encephalitis included a 5-year-old boy with pre-existing motor tics that worsened during the encephalitic process [15]. The tics were associated with emotional dysregulation and obsessionality. In this report, the definition of basal ganglia encephalitis included positive serum dopamine-2-receptor autoantibodies.

### Autoimmune encephalitis

Autoimmune encephalitis is a heterogeneous condition in which antibodies target neuronal surface proteins. The presence of tics in the context of autoimmune encephalitis has been associated with antibodies binding to several targets, including *N*-methyl-d-aspartate receptor (NMDAR), extracellular voltage-gated potassium channels (VGKC), and glycine receptor (GlyR) [46, 47]. The importance of identifying autoimmune encephalitis as a cause of tic disorders should not be underestimated, because these disorders are often amenable to treatment with immune modulation therapy [48]. Furthermore, selected cases of autoimmune basal ganglia encephalitis have been categorised as encephalitis lethargica [49].

#### Anti-NMDAR encephalitis

Anti-NMDAR encephalitis is a severe form of autoimmune encephalitis associated with antibodies against the NR1 and NR2 subunits of the NMDA receptor [48]. Anti-NMDAR encephalitis is often related to an underlying malignancy, especially ovarian teratoma. Compared to basal ganglia encephalitis, anti-NMDAR encephalitis is more prevalent in females and is more frequently associated with orofacial dyskinesias and stereotypies [50]. It has been suggested that young patients with anti-NMDAR encephalitis tend to have a different clinical presentation compared to adults: hyperkinetic manifestations (especially orofacial dyskinesias, choreoathetosis, oculogyric crises, dystonia, and stereotypies) are consistently reported in children, whereas psychiatric manifestations, such as acute psychosis, are more frequently diagnosed in adults [48, 51]. Of note, tics are often absent in descriptions of anti-NMDAR encephalitis from large studies of patients presenting with hyperkinetic manifestations [52, 53].

Nevertheless, in a retrospective study of Malaysian children with anti-NMDAR encephalitis, 2 out of 11 patients presented with dystonic tics [16]. Moreover, two individual case reports mentioned tics as a presenting feature of anti-NMDAR encephalitis in an 11-year-old girl with motor and phonic tics associated with an ovarian teratoma [17] and a 4-year-old Lebanese child with phonic tics associated with orofacial dyskinesias and gait problems [20]. These observations raise the possibility that tics might have been under-diagnosed or overlooked in anti-NMDAR encephalitis.

#### Voltage-gated potassium channel complex (VGKC) encephalitis

VGKC encephalitis is a relatively recently recognised autoimmune disorder associated with antibodies against specific subunits of the VGKC complex. Although the clinical presentation of this condition is highly variable, neuropsychiatric symptoms including cognitive impairment, seizures, and behavioural problems have frequently been described [46]. The pathophysiology of VGKC encephalitis is characterised by a highly heterogeneous nature and reports of tics are rare. Moreover, VGKC encephalitis has been investigated more extensively in adults, and there are limited studies in children [54]. In a retrospective case series of 12 children with VGKC encephalitis and heterogenous pathophysiology, half of them had a movement disorder and one child presented with motor tics [18]. A case report described a 13-year-old male with positive anti-VGKC antibodies associated with behavioural changes, language difficulties, and amnesia developed 2 weeks after a mild flu-like illness. Interestingly, he also presented with multiple movement disorders, including violent cervical tics, bradykinesia, choreoathetosis of his upper limbs, ballismus of his lower limbs, and facial dystonia [19]. A 72-year-old Hispanic female was described as a case of VGKC encephalitis mimicking sporadic Creutzfeldt-Jakob disease: she presented with orofacial tics, hypertonia, dysmetria, echolalia, and visual and auditory hallucinations [22].

#### Anti-glycine receptor (anti-GlyR) encephalitis

Anti-GlyR antibodies were first documented in 2008 in a case of progressive encephalomyelitis with myoclonus and rigidity [55]. They have subsequently been recognised and documented in other clinical presentations. A case report presented a previously healthy 40-year-old male who tested positive for anti-GlyR antibodies after a 5-day history of upper respiratory tract infection, evolving into difficulties with breathing and swallowing plus involuntary jerking. This patient reported a 1-week prodrome of involuntary tic-like jerks, affective symptoms, and hallucinations [21]. Interestingly, tics were also reported in a more recent case of anti-GlyR antibodies from Australia [23].

### Post-infectious encephalitis

Pathogenic invasion of the central nervous system can result in a range of movement disorders, from parkinsonism to chorea, as certain neurotropic viruses have an affinity specifically for the basal ganglia [56]. Sporadic cases of infectious processes leading directly to encephalitis and concurrent tics have also been documented. In some cases, the presence of tics cannot be confidently associated with the encephalitic episode due to the presence of confounding structural pathology. For example, a 1996 cohort study of patients with obsessive-compulsive disorder included a case of childhood post-encephalitis hydrocephalus (pathogen undocumented) that resulted in adult-onset compulsive behaviours as well as multiple motor and phonic tics [25]. Overall, both the infectious agent and the presentation of the movement disorder show a wide variability, depending on the geographical location. For example, studies conducted in India have shown a higher prevalence of rheumatic fever and tetanus [57] compared to cases of *Mycoplasma pneumoniae* infection leading to motor abnormalities in both the USA [28] and Japan [58].

#### Viral infections

Various neurotropic viruses have been implicated in the aetiology of encephalitis leading to tics. These include the human immunodeficiency virus (HIV), herpes simplex virus (HSV), varicella zoster virus (VZV), the flavivirus family (West Nile virus encephalitis and Japanese encephalitis), measles, mumps, and rubella.

##### HIV

Movement disorders are a relatively rare complication in patients with HIV infection; however, their incidence has been shown to increase with disease progression [59]. Direct inflammation resulting from the viral infection (HIV encephalitis), opportunistic infection, and medication adverse effects are recognised as the main aetiological factors [60]. The most commonly reported movement disorders in patients with HIV are tremor, hemichorea, and hemiballismus [60]; however, there have been sporadic case reports of tics. In rare cases, movement disorders can be the first presentation of HIV infections. In a case series by Mirsattari et al., a previously healthy man who presented with facial tics, dystonia, and torticollis, among other hyperkinetic manifestations, was subsequently diagnosed with an HIV infection [26]. In a more recent case report, a 28-year-old woman diagnosed with HIV presented to hospital 4 months later with loss of consciousness, hemichorea, and motor tics affecting her face [35]. Interestingly, this patient was diagnosed with toxoplasma encephalitis based on imaging and clinical findings. These findings suggest that encephalitis should be excluded in adults developing new-onset tics in the presence of underlying risk factor, such as immunosuppression. In terms of pathophysiology, it has been suggested that the HIV infection can directly affect basal ganglia dopaminergic pathways [61]; however, the exact pathological mechanisms through which the HIV infection can result in encephalitis are still unclear.

##### Herpes simplex virus (HSV)

HSV encephalitis is a relatively common condition, accounting for approximately 10% of all cases of encephalitis worldwide [62]. The neurological complications of HSV encephalitis in children are known to include choreoathetosis, orofacial dyskinesias, and tics [63]. However, the pathophysiological processes linking HSV encephalitis to movement disorders are poorly understood. It has been proposed that direct viral damage to the basal ganglia and secondary autoimmune response involving anti-NMDAR antibodies could play key roles [64]. For example, in a case series of 8 children with neurological complications secondary to HSV encephalitis, 63% of patients had anti-NMDAR autoantibodies [65]. An earlier case report of a 6-year-old girl with eye blinking, grimacing, and head twitching, as well as complex motor and phonic tics, which developed 2 weeks after recovering from HSV encephalitis [30]. All tics responded well to treatment with antidopaminergic medication. Neuroimaging revealed oedema of the right basal ganglia and the authors associated the development of tics with the pathological involvement of the limbic system and/or basal ganglia.

##### Varicella zoster virus (VZV)

Varicella zoster virus (VZV) is the second most common cause of encephalitis in the UK, following HSV [66]. Movement disorders have been documented as possible sequelae of VZV [66]. A case report documented tics in a 4-year-old boy who was suffering from an encephalitis secondary to VZV infection [31]. The patient presented with alterations of consciousness and tremor and was diagnosed with parainfectious encephalitis. Although his initial symptoms resolved, he subsequently developed a chronic motor tic disorder involving his face and neck. During the acute encephalitis, brain magnetic resonance imaging demonstrated hyperintensities at the level of the striatum, which suggests a possible role for the basal ganglia in the pathophysiology of the tic disorder, although the neuroimaging changes and tic onset were not temporally linked.

##### Flaviviruses

Flaviviruses are single-stranded RNA enveloped viruses and are clinically relevant due to their global presence and plethora of symptoms [67]. Japanese encephalitis and West Nile virus encephalitis have been linked to hyperkinetic manifestations, including secondary tics. Movement disorders that have most commonly been reported in patients with Japanese encephalitis include parkinsonism, myoclonic jerks, and oculogyric crises [68]. A retrospective cohort study investigated the outcomes of 68 patients 5 years after the resolution of the acute infection and found that 7 patients experienced motor tics, along with chorea and athetosis [24]. West Nile virus, despite being symptomatic in only 20% of those who are infected, can result in central nervous system pathology. Francisco et al. studied the phenotype of Californians who tested positive for West Nile virus in 2004 and identified 5 paediatric cases who experienced encephalitis [27]. One of these patients, who had a history of acute lymphoblastic leukaemia, developed facial tics and athetosis 3 weeks following the acute encephalitis. These findings confirm the relatively infrequent reporting of tics in patients with West Nile virus encephalitis.

##### Measles, mumps, and rubella

The incidence of measles, mumps, and rubella has declined due to the introduction of vaccination programmes across the world. Despite this, important complications of these viruses, which include encephalitides, are intermittently reported [69]. A case report from India described the onset of encephalitis 8 years following measles, which presented with motor tics [34]. This patient was a 10-year-old boy who experienced complex shoulder and facial tics with insidious onset. He was initially diagnosed with a primary tic disorder and subsequently experienced a significant worsening of symptoms, including ataxia and myoclonus. Thorough investigations revealed positive serum and cerebrospinal fluid measles IgG, and the patient was diagnosed with subacute sclerosing panencephalitis. The patient’s condition gradually improved over 6 months with a combination of antiviral and antiepileptic medication. In a previous report of subacute sclerosing panencephalitis, cognitive decline was associated with involuntary movements that could not be confidently labelled as tics [70].

Tics have also been reported as a result of mumps infection in a 9-year-old boy who developed multiple motor and phonic tics 2 months after recovery from mumps complicated by meningoencephalitis [32]. The tics were pronounced and included eye blinking, finger flexing, and throat clearing. Treatment with risperidone led to a considerable improvement in tic severity. Magnetic resonance imaging revealed no anatomically significant lesions which could explain the movement disorder.

As part of a large retrospective study, Mejia et al. identified a girl who experienced sudden-onset, severe motor and phonic tics associated with gait problems, following rubella infection at the age of 8 [28]. As an adult, the patient had brain magnetic resonance imaging showing bilateral lesions on the putamen and globus pallidus, raising the possibility of a link between her rubella infection and neuroimaging abnormalities.

#### Non-viral infections

The previously discussed viruses are typically associated with encephalitis due to their neurotropic nature [1]. However, there are also documented cases of tic disorders through central nervous system invasion by non-viral agents in Creutzfeldt-Jakob disease (CJD), neurobrucellosis, and *Mycoplasma pneumoniae*-associated encephalitis.

##### Creutzfeldt-Jakob disease (CJD)

CJD is a prion disorder that typically presents with rapidly progressing cognitive decline, ataxia, and personality change, sometimes associated with catatonia and mutism [33]. A 59-year-old female presented with classic CJD symptoms which developed over the course of 5 months and led to akinetic mutism superimposed by phonic tics and involuntary movements of the limbs [33]. The phonic tics included grunting and barking, whereas the involuntary movements were similar to those reported by patients with anti-NMDAR encephalitis (grimacing, eye movements) [48, 52, 71]. VGKC encephalitis has also been shown to mimic features of CJD [22], whereas catatonic features have been reported in both CJD and GTS [42]. However, the patient reported by Molina et al. met the diagnostic criteria for sporadic CJD based on both clinical and electroencephalographic findings [33].

##### Neurobrucellosis

Neurobrucellosis is an infective condition caused by the zoonotic bacterium *Brucella*, potentially resulting in inflammation of the central nervous system with abscess formation. A retrospective observational study, conducted in Turkey, where brucellosis is endemic, identified paediatric patients with neurobrucellosis. One of these patients presented with a chronic cough that was initially classed as a pulmonary manifestation of brucellosis and later diagnosed as a phonic tic [29]. Positive *Brucella* IgM antibodies and full recovery with anti-brucellosis treatment confirmed the diagnosis.

##### *Mycoplasma pneumoniae*

*Mycoplasma pneumoniae-*associated encephalitis is not rare in the paediatric population; however, its exact pathophysiological mechanism is still debated, largely because of inconsistent laboratory findings [72]. An 18-year-old male patient from a large retrospective study by Mejia et al. developed sudden onset ataxia and simple motor tics following *Mycoplasma pneumoniae* infection [28]. Magnetic resonance imaging at the time of presentation showed pathological changes in the striatum, which resolved with successful patient recovery, suggesting a possible pathophysiological role.

## Conclusion

The clinical presentation of encephalitis varies according to its aetiology, as well as the geographical and demographic characteristics of the patients [11]. Movement disorders are common manifestations of encephalitis in autoimmune and post-infective illnesses, as well as specific encephalitis of unknown aetiology (e.g. encephalitis lethargica). However, there is a relative paucity of literature focusing on tics in patients diagnosed with encephalitis. Our systematic literature review found that tics are reported less frequently than other dyskinesias, such as chorea and myoclonus. This might reflect a genuine absence of tics in the majority of patients with acute encephalitis and in post-encephalitic states. However, there is also the possibility that tics are under-reported in the literature. A common theme found in the reviewed literature was the heterogeneous terminology used to describe abnormal movements in post-encephalitic patients, which includes ‘tic-like movements’ and ‘non-specific dyskinesias’ [21]. Other factors could have contributed to the possible under-recognition of tics. For example, it has been found that stereotypies (hyperkinetic symptoms that can be difficult to differentiate from tics) are often a manifestation of encephalitis in children [73]. Additional uncertainties arise in regard to the widespread reporting of oculogyric crises in patients with encephalitis lethargica [37]: despite being an established feature of the pandemic, oculogyric crises can pose significant difficulties in the differential diagnosis with ocular tics [74]. Likewise, it has been highlighted that the differential diagnosis between tics and focal epileptic seizures can be challenging, especially in patients with lesional epilepsy due to underlying pathology resulting in other cognitive and neuropsychiatric symptoms [75]. In the reviewed literature, motor abnormalities were not consistently assessed by movement disorder specialists and sometimes the diagnostic process relied on video footage, with minimal clinical detail. Overall, a number of relevant studies and reports lacked clinical detail about tics, possibly they were not the main research focus. A lack of clinical detail was frequently encountered in cases from the encephalitis lethargica pandemic, which were mainly extracted from the historical literature. The categorisation of different types of encephalitis based on aetiology also posed specific problems. For example, due to the unknown aetiology of encephalitis lethargica, some presumed cases are classed as autoimmune basal ganglia encephalitis in the existing literature. Similarly, there is an established overlap between autoimmune (e.g. anti-NMDAR) and infective (e.g. CJD) causes of encephalitis. Finally, the reliance on anecdotal case studies makes it difficult to quantitatively analyse the presence of tics in a large cohort of patients with encephalitis.

Despite these limitations, the first literature review evaluating the presence of tics in reported cases of encephalitis showed that tics have been sporadically reported in cases of encephalitis resulting from autoimmune, infective, and unknown aetiology. Specifically, tics have been more commonly reported in the post-encephalitic period and involvement of the basal ganglia was frequently found. Furthermore, the association of new-onset tics and encephalitis, in the background of other neuropsychiatric abnormalities, has clinical implications in potentially improving the detection of encephalitis based on clinical features. Future research should focus on the categorisation and treatment of hyperkinetic movement disorders associated with encephalitis.
